# Extensively coated revision stems in proximally deficient femur: Early results in 15 patients

**DOI:** 10.4103/0019-5413.39554

**Published:** 2008

**Authors:** SKS Marya, R Thukral

**Affiliations:** Max Institute of Orthopedics and Joint Replacement, Max Super Specialty Hospitals, 1, Press Enclave Road, Saket, New Delhi - 110 017, India

**Keywords:** Cementless fixation, extensively coated, proximally deficient femur

## Abstract

**Background:**

Hip replacement following failed internal fixation (dynamic hip screw for intertrochanteric fractures) or previous hip arthroplasty presents a major surgical challenge. Proximal fitting revision stems do not achieve adequate fixation. Distal fixation with long-stemmed extensively coated cementless implants (like the *Solution*™ system) affords a suitable solution. We present our early results of 15 patients treated with extensively coated cementless revision stems.

**Materials and Methods:**

Fifteen patients with severely compromised proximal femora following either failed hip arthroplasty or failed internal fixation (dynamic hip screw fixation for intertrochanteric fractures) were operated by the senior author over a two-year period. Eight patients had aseptic loosening of their femoral stems following cemented hip replacements, with severe thinning of their proximal cortices and impending stress fractures. Seven had secondary hip arthritis following failure of long implants for comminuted intertrochanteric or subtrochanteric femoral fractures. All patients were treated by removal of implant (cemented stems/DHS implants) and insertion of long-stemmed extensively coated cementless revision (‘*Solution*™ *DePuy, Warsaw (IN), US*’) stems along with press-fit acetabular component (Duraloc Cup, DePuy, Warsaw (IN), US). All eight hip revisions needed extended trochanteric osteotomies.

**Results:**

All patients were primarily kept in bed on physiotherapy for six weeks and then gradually progressed to weight-bearing walking over the next six to eight weeks. The Harris Hip Scores and patient satisfaction were used for final evaluation. We achieved good results in the short term studied. In our first three patients (all following failed cemented total hip replacements), we resorted to cerclage wiring to hold osteotomised segments (done to facilitate stem removal). The subsequent 12 proceeded without the need for cerclage wiring. One patient had a intraoperative severe comminuted fracture extending into the supracondylar region while hammering in the stem. Post cerclage wiring, she was put on a long knee brace and her mobilization was delayed to 12 weeks.

**Conclusions:**

The extensively coated cementless (‘*Solution*™’) femoral stem provides a reasonable ‘solution’ to the deficient femur in hip revision. The proximal femoral deficiences can be relatively easily bypassed and distal fixation can be achieved with this stem. Extreme care needs to be taken to avoid fractures and penetration of the femoral shaft, which can, however, be managed by cerclage wiring. Principles of a successful outcome include preservation of the functional continuity of the abduction apparatus, care to recognize and prevent distal extension of fracture while inserting the stem (preemptive cerclage wiring) and supervised rehabilitation.

## INTRODUCTION

Total hip arthroplasty (THA) offers a reliable treatment option that relieves pain and improves function in elderly patients with end-stage arthritis of the hip. Middle-aged and young patients with hip arthritis, however, present a challenge because their expected lifespan is long and in general, the results of THA are time-limited. As the frequency of primary THA increases, the possibility of high revision incidence rates within the next decade seems very real.

Over the last generation, arthroplasty surgeons have repeatedly utilized cement as the grout for both the acetabulum and femur. When considering having THA surgery for the second or third time, concerns arise about the outcome. Revision increases the surgical and medical challenges. With revision, higher rates of second and third revisions (re-revisions), periprosthetic fractures, dislocations and septic and aseptic osteolysis are expected.

Total hip arthroplasty done with cemented femoral components have shown long-lasting reproducible results in the elderly population, but younger patients often have poor results.^1^–^2^ In an attempt to improve longevity, particularly in younger patients, cementless femoral components have been used. The majority of these devices have a porous coating on their surface that allows for the ingrowth of bone and fibrous tissue into its interstices. It is hypothesized that this method of fixation allows for a more durable reconstruction than offered by cementation. There are devices that have porous coating only on the proximal portion of the stem and gain purchase in the femoral methaphysis and there are extensively coated stems that obtain purchase more distally at the femoral isthmus - all of these provide ‘biologic fixation’.

Revision hip replacement in proximally compromised femurs presents a significant surgical challenge. Proximal fitting revision stems do not achieve adequate fixation. Distal fixation with long-stemmed extensively coated cementless implants like the *Solution™* stem (DePuy, Warsaw [IN], US revision hip system) affords a viable solution. However, in femoral stems with extensive proximal deficiency (such that distal scratch fit of 5-7 cm is not possible) or with patulous medullary canals, viz. Paprosky Type IIIb and IV defects,^3^ one may have to consider alternatives.

We present here our short-term results of 15 patients with proximally deficient femora (following failed cemented total hip replacements or failed internal fixation for intertrochanteric fractures) treated with distal fixation (using extensively coated cementless revision stems).

## MATERIALS AND METHODS

Between April 2003 and March 2005, 15 consecutive hip arthroplasty procedures using extensively coated femoral components were performed in 15 patients (eight women and seven men). The mean patient age at operation was 53.4 years (range, 45-65 years). In the initial surgery, eight of the hips had been diagnosed as hip osteoarthritis, primary or secondary, for which they had undergone cemented THA. The primary diagnosis of the remaining seven was a intertrochanteric fracture fixed with dynamic hip screw (DHS) with either implant failure or secondary hip arthritis.

In the eight hips that underwent revision surgery, the diagnosis was aseptic loosening of the previous femoral component (all cemented THAs) with severe thinning of the proximal femoral cortices and radiological evidence of impending fractures. The seven DHS-fixed hip fractures had failed by implant cutout and had an unsalvageable femoral head in four cases, nonunion in two cases and avascular necrosis, with subsequent secondary hip osteoarthritis in one case. All patients had additional co-morbid medical factors, including diabetes mellitus (seven patients), hypertension (12 patients), renal disease (one patient), ischemic heart disease (two patients) and chronic obstructive airway disease (two patients).

Preoperative radiographs of the pelvis and femur (anteroposterior and lateral views) were templated to estimate the length and diameter of the stem in order to obtain a scratch fit between 4 and 6 cm of the cortical bone. All operations were performed through the anterolateral approach. All eight hip revisions needed extended trochanteric osteotomies (trans-trochanteric approach) for the exposure or removal of the failed component or cement.^4^–^5^ Trochanteric cerclage wiring was used to repair osteotomy sites after insertion of the stem in these patients.

A straight femoral component was used in all cases - ‘*Solution™*’ a modular femoral component (DePuy, Warsaw [IN], US), that has extensive porous coating, modular, with a 28-mm head. The most commonly used components were 200 mm long (*n* = 14) and 13.5 mm in diameter (*n* = 10). Other components used were the straight 135 mm long (*n* = 1) and 15 mm diameter (*n* = 4), 16.5 mm diameter (*n* = 1) stems. The acetabular component was also press-fit. We used the Duraloc cup (DePuy, Warsaw [IN], US) in all cases and adjunctive fixation was achieved with screws (needed in six cases, all of them revised from primary cemented THAs). No antiprotrusio ring was required.

Patients were evaluated preoperatively and postoperatively using Harris Hip Scores (with special emphasis on the ability to walk unaided without a limp) and patient satisfaction with the procedure using a visual analog scale [VAS] model. Patients were directly questioned at the most recent visit for the presence or absence of thigh pain.

Radiographs at each late review included anteroposterior (AP) and lateral views of the femur and these were compared with radiographs obtained six weeks postoperatively. Patients were reviewed at six weeks, three months, six months, 12 months and 24 months postoperatively. All patients were primarily kept on in-bed mobilization for six weeks and then gradually progressed to weight-bearing walking over the next six to eight weeks.

Demographic factors, operative details, Harris hip scores before the revision and at final follow-up and postoperative thigh pain, if any, were recorded. Postoperative radiographs were studied to determine lysis or loosening. The criteria used for evaluation included the Harris hip scores at final follow-up, as well as overall patient satisfaction (on a visual analog scale model). Results were classified into excellent, good or poor on a simplified assessment scale (taking any improvement from the preoperative highest Harris hip score and minimum 5 points on the VAS satisfaction score as a good result and extrapolating excellent and poor results) as depicted in Tables 1 and 2.

**Table 1:** The details of the patients analysed

Patient	Age/Sex	1° diagnosis	1° implant	Pre-op score	Duration of Sx	Screws in cup	Blood loss	Complications	Wt bearing
JMS	78/M	AVN	THR	51	1hr 40m	No	450ml	Split #	5mths
SLJ	55/F	AVN	THR	52	1hr 55m	No	550ml	Split #	5½mths
FMS	62/M	# NOF	THR	55	1hr 40m	No	650ml	Split#	5mths
JKR	56/M	# IT	DHS	32	1hr 25m	No	250ml	-	3mths
MPK	72/F	# IT	DHS	24	1hr 30m	No	350ml	-	4mths
MSM	61/F	# subtroch	DHS	29	1hr 40m	No	450ml	-	5mths
RS	69/F	RA	THR	41	2hr 25m	Yes	650ml	Shaft #	8½mths
DM	37/M	AVN	THR	37	1hr 55m	No	750ml	-	5mths
MSM	54/M	# subtroch	DHS	42	1hr 20m	No	250ml	-	4mths
YM	72/F	# IT	DHS	39	1hr 10m	No	250ml	-	3½mths
RC	52/F	RA	THR	35	1hr 30m	Yes	450ml	Sup. Inf.	5mths
AG	55/M	# NOF	DHS	32	1hr 20m	No	300ml	-	4mths
JM	49/M	# IT	DHS	34	1hr 10m	No	400ml	-	3mths
PN	69/F	OA	THR	32	1hr 40m	No	550ml	-	5mths
KSR	75/M	# NOF	THR	30	1hr 45m	No	650ml	-	5½mths

# Fracture, NOF - Neck of femur, AVN-Avascular necrosis, IT - Intertrochanteric, RA - Rheumatoid arthritis, OA - Osteo arthritis

**Table 2:** Criteria for result evaluation and our results

Result	Pre-op harris score	Post-op harris score	Patient satisfaction	Patients
Excellent	< 40	> 75	> 7	3
Good	< 40	50-75	5-7	11
Poor	< 40	< 50	< 5	1

## RESULTS

The Harris Hip scores improved from a preoperative average of 38 (range, 24-51), to a postoperative average of 71 (range, 21-91). Patient satisfaction scores similarly improved from a preoperative average of 5, to a postoperative average of 7.

**Figure 1:**
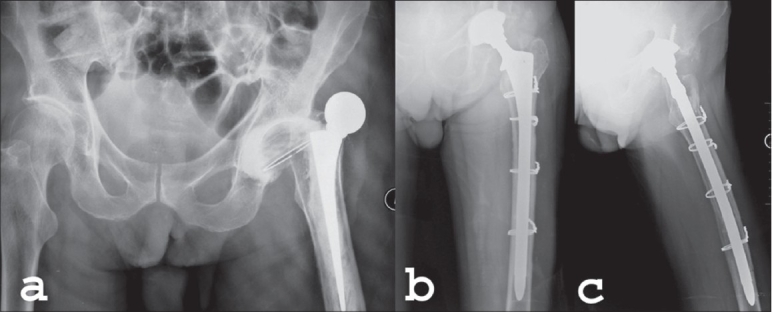
(a) Preoperative X-Ray (AP view) of the pelvis shows three-week-old dislocated retroverted cemented total hip arthroplasty (THA). (b) Postoperative X-Ray (AP and lateral) of left hip at 11 months of cementless revision stem (‘Solution’™) for the failed THA

**Figure 2:**
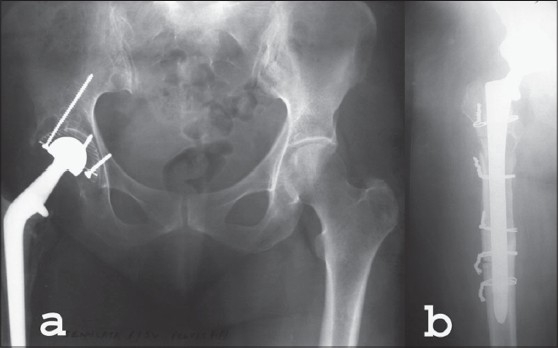
(a) Preoperative X-Ray (AP view) of a patient operated 20 years ago with painful loose cemented right THA. (b) Postoperative X-Ray (AP) of right hip of the same patient at four months of cementless revision stem (‘Solution’™)

**Figure 3:**
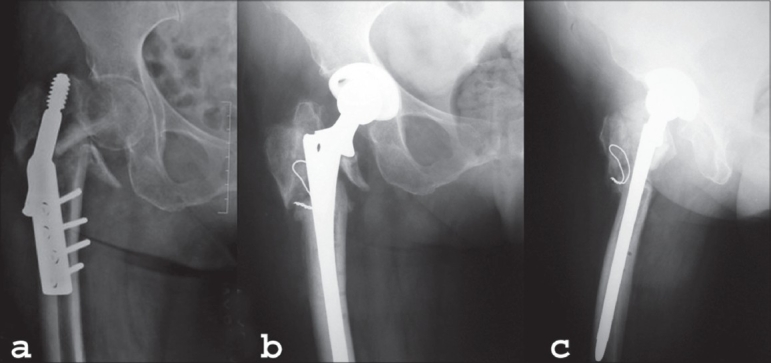
(a) Preoperative X-Ray (AP view) of right hip shows failed DHS for intertrochanteric fracture femur. (b) Postoperative X-Ray (AP and lateral view) of the same hip at 15 months of cementless revision stem (‘Solution’™)

In our first three patients (all following failed cemented total hip replacements), we resorted to extended cerclage wiring to hold osteotomised segments (done to facilitate stem removal) [Figures 1 and 2]. The subsequent 12 (five post-THA and seven post-failed DHS) proceeded without the need for extensive cerclage wiring [Figure 3]. There was no need of osteotomy in the seven cases following DHS removal, but during revision with cementless stems, there is a risk of propagating a fracture from the trochanter down into the shaft, which luckily did not happen in our cases and so cerclage wiring was not required. One patient had a severe comminuted fracture and extension of this fracture distally (to the supracondylar region) intraoperatively while hammering the stem. Post cerclage wiring, she was put on long knee brace and her mobilization was delayed. One other patient developed superficial infection which healed without sequelae. There was no limb length discrepancy, measured or felt by any patient following the cementless stems. In cases of previous scars, we followed the same incision, if sandard incision was falling on the previous scar or else ignored and proceeded with our independent incision. There were difficulties encountered in removing distal plug, so we had to extend our osteotomy to the level of the plug. However there wasn't any significant difference between the two groups. The preoperative and intraoperative details, as well as distribution of our results have been summarized in the tables. We achieved excellent and good results in 3 and 11 respectively.

## DISCUSSION

Revision hip replacement presents its own unique set of challenges. One of the issues that need to be addressed includes the method of femoral stem fixation (cemented or cementless). Amongst the cementless group, further decision needs to be taken as to the extent of coating (proximal, distal or extensive) and stability (primary press-fit and/or secondary bony ingrowth^6^). The possible complications (fractures, stress shielding, osteolysis, loosening, subsidence and migration) and prognosis also needs to be looked into.

The method of choice of fixation of the revised femoral components is controversial. Despite improvements in cementing techniques, the re-revision rates of cemented femoral components still increase with time and the radiological loosening rates are still high.^7^ Other disadvantages cited are extensive bone loss (to accommodate cement) and inadequate primary (inability to achieve proximal stability with deficient femora) or secondary stability (windshield wiper loosening of the cemented stem). Some authors^8^ have, however, reported good results when cementation of the femur was used in conjunction with impacted cancellous allografts. This method is however technically demanding and fraught with complications (fracture and early subsidence).^8^

Proximally porous-coated femoral components have not known to consistently produce favorable outcomes.^9^–^10^ Increased interest in proximally porous coated stems was sparked in the 1980s by first generation cemented stem failures in young patients and concerns arising due to extensively porous coated cementless stems regarding thigh pain and stress shielding. Despite design modifications, the modern proximally coated stem has not eliminated thigh pain or stress shielding.^9^ Highly modular designs that afford assembly of a combination of adequately filling metaphyseal and diaphyseal portions may work to achieve the goals of appropriate primary as well as secondary fixation and stability.^11^ The intraoperative flexibility provided by choices of diameter, stem length, fixation type and proximal stem size and orientation is purported to enable establishment of a stable hip center.^11^ This however, needs to be customized to each revision situation. However, only a few short-to intermediate-term results have been reported.^12^–^13^ Proximal coating does not protect against loss of bone mineral content proximally or distally in the femur. Decreasing the extent of porous coating alone does not necessarily reduce proximal femoral bone loss.^6^,^14^

Isolated distal coated implants have been reported to show extensive proximal stress shielding and osteolysis with trochanteric fractures.

Extensively porous-coated femoral components with distal bone fixation as the primary fixation principle have shown promising results in numerous long-term studies,^9^,^15^–^17^ both clinically and radiographically. The components can achieve secondary stability by distal bone ingrowth where the condition of the host bone is still good, more so when the quality of the proximal bone stock is poor.^9^,^17^ The Wagner prosthesis has been suggested as an attractive option, because it can restore the proximal bone stock. Subsidence of the component, cost considerations and high rates of dislocation may, however, preclude its more extensive use.^18^ The clinical results of a series using an extensively Hydroxyapatite (HA) coated stem were similar to those using an extensively porous-coated stem. So the question of whether an extensively HA-coated implant will be superior to an extensively porous-coated implant with regard to stress shielding remains as yet unanswered.^19^

Stability all along the stem is desirable. Boden *et al.*,^20^ demonstrated in their radiological study on periprosthetic bone changes in two different uncemented femoral stems employing different concepts of fixation that, unstable stems eventually led to loss of bone mineral density and eventual loosening along the entire length of the stem, leading to early loss of fixation and failure.^6^,^20^

Stressshielding has not shown to produce adverse consequences in extensively porous coated THAs. A long-term study^21^ on the clinical consequences of stress shielding in a series of 223 cementless THAs compared the outcome of 48 THAs that had radiographically evident stress-shielding with 160 THAs that did not have radiographically visible stressshielding or that had less severe stressshielding. Stress-shielding was found to be more likely in females, patients with a low cortical index and patients with larger stems. Patients with stressshielding had a lower mean walking score than patients without stressshielding and less osteolysis.^21^ No patient with stressshielding, however, had any loosening, implant fractures or loss of porous coating. The revision rate was 13% (six hips) among hips with stressshielding and 21% (33 hips) among hips without stressshielding. Fifteen-year survival was 93% among hips with stressshielding and 77% among hips without stress-shielding.^21^ Severe stressshielding correlates with preoperative osteoporosis and larger diameter stems but not necessarily failure.^15^,^22^

In a postmortem study^14^ evaluating the level of proximal femoral remodeling by varied degrees of porous coating, the extensively coated group showed less bone loss on average (18.4%) than the proximally coated group (38.6%). Further, there was no relationship between the extent of coating and the location of bone mineral loss.^14^

Persistent thigh pain has been cited as one of the most disabling complications following cementless femoral fixation. Reasons cited by various authors have included stem tip cortical hypertrophy, stress fractures and intermittent impingement (inadequate distal fill). Paprosky *et al.*,^23^ reported in their study of 170 patients that after a mean follow-up of 13.2 years, the total mechanical failure rate was only 4.1%. Bone ingrowth was achieved in the majority (83%) of patients. Only one patient experienced considerable thigh pain but this spontaneously subsided with time. The high incidences of thigh pain reported may be related to the larger size of the femoral component used and distal canal impingement that was achieved. Eighty-five per cent of the femoral components used had a diameter of 13.5 mm or more in his series.^23^ Significant thigh pain in bone ingrown stems has been observed more commonly in osteoporotic femurs and bone stock deficient femurs.^22^

Intraoperative fractures have been reported by many authors with different implant designs and approaches. Caution needs to be exercised when inserting a long, straight, extensively coated femoral component. Paprosky *et al.*,^23^ reported intraoperative fractures during stem insertion in 8.8% of patients in their series; however, the predisposing factors to this complication were not mentioned. In our study, there were three intraoperative controlled fractures and one distal extension with perforation. We tried to correlate our cases with diaphyseal perforation and distal extension of the fracture with the use of a straight non-anatomic long and thick femoral stem. A radiographic study^24^ found that significant anterior cortical thinning was more common in Chinese patients if 200-mm straight stems were used,^25^ attributed to the more pronounced anteroposterior bowing of femora in the Chinese population.^24^–^25^ We have used the bowed 200-mm femoral components and although our experience is very limited, they have shown to help minimize the risk of anterior cortical erosion or distal perforation.

The use of a strut allograft in conjunction with an extended trochanteric osteotomy in patients with poor proximal femoral bone stock decreases the stresses on the stem by 48% and has been recommended by Busch *et al.*,^26^ in their analysis of fractures in distally fixed femoral stems.

Our series has a very short follow-up to really determine the true efficacy of this cementless system in the long term, especially with regards to proximal stress shielding and osteolysis. However, all but one patient had considerable improvements in their hip scores and were very uniformly satisfied with the procedure at last follow-up. Traditionally, long-stem cemented femoral stems were used to tackle the problem of proximal bone deficiency with uniformly poor results.^7^–^8^ We may be premature in concluding that the extensively coated revision femoral stem works wonderfully in bypassing proximal femoral deficient femora, but in the short term, our patients have shown results comparable to results following cementless hip revisions in other centers worldwide.^9^,^17^ The possibility of secondary stress shielding leading to proximal osteolysis also seems remote theoretically (as secondary bone ingrowth has been shown) with extensive coating.

It must, however, be emphasized that principles of a successful outcome in such a scenario include preservation of the functional continuity of the abduction apparatus during surgery, care to recognize and prevent distal migration of fracture while inserting the stem (by preemptive cerclage wiring) and supervised rehabilitation.

## CONCLUSIONS

The extensively coated cementless (*‘Solution’*) femoral stem may provide a reasonable ‘solution’ to this extremely challenging situation. The proximal femoral deficiencies can be relatively easily bypassed and distal fixation achieved with this porous-coated stem. However, extreme care needs to be taken to avoid fractures and penetration of the femoral shaft (which can occur if a straight stem is inserted without understanding the natural bowing of the femoral shaft). This can be managed to a great extent by cerclage wiring.
